# Architectural delineation and molecular identification of extracellular matrix in ascidian embryos and larvae

**DOI:** 10.1242/bio.026336

**Published:** 2017-09-15

**Authors:** Jiankai Wei, Guilin Wang, Xiang Li, Ping Ren, Haiyan Yu, Bo Dong

**Affiliations:** 1 Ministry of Education Key Laboratory of Marine Genetics and Breeding, College of Marine Life Sciences, Ocean University of China, Qingdao 266003, China; 2 Institute of Evolution and Marine Biodiversity, Ocean University of China, Qingdao 266003, China; 3 Laboratory for Marine Biology and Biotechnology, Qingdao National Laboratory for Marine Science and Technology, Qingdao 266237, China

**Keywords:** *Ciona*, Extracellular matrix, Collagen, RNA sequencing

## Abstract

The extracellular matrix (ECM) not only provides essential physical scaffolding for cellular constituents but also initiates crucial biochemical and biomechanical cues that are required for tissue morphogenesis. In this study, we utilized wheat germ agglutinin (WGA) staining to characterize the ECM architecture in ascidian embryos and larvae. The results showed three distinct populations of ECM presenting in *Ciona* embryogenesis: the outer layer localized at the surface of embryo, an inner layer of notochord sheath and the apical ECM secreted by the notochord. To further elucidate the precise structure of *Ciona* embryonic ECM, we employed scanning and transmission electron microscopy, and found that the outer membrane was relatively thick with short fibres, whereas the ECM layer in notochord sheath was not as thick as the outer membrane but more regular arranged; the lumen between notochord cells was hydrostatic and sticky. Then, we used the RNA sequencing data from the embryos and larvae of *Ciona savignyi* to identify ECM genes and acquire their expression patterns. We identified 115 unigenes as 67 ECM genes, and 77 unigenes showed dynamic expression changes between different stages. Our results reveal the architecture, molecular composition and dynamic expression profile of ECM in ascidian embryogenesis, and may increase understanding of the function of the ECM in chordate development.

## Abstract (precis)

**Summary:** This study reveals the architecture, molecular composition and dynamic expression profile of the extracellular matrix in ascidian embryos and larvae, providing clues for its function in chordate development.

## INTRODUCTION

The extracellular matrix (ECM) is the fundamental cellular component of multicellular organisms. It is secreted from and distributes on the cell surface, providing not only essential physical scaffolding for diverse cellular processes, but also crucial biochemical and biomechanical cues that are required for tissue morphogenesis and homeostasis (Frantz et al., 2010).

The ECM is composed of three main classes of macromolecules: collagens, proteoglycans and glycoproteins. Collagen molecules often form a triple-stranded helix that can subsequently assemble into supramolecular complexes, such as fibrils and networks. To date, 28 types of collagens and >40 distinct α-chains have been identified in vertebrates (Ricard-Blum, 2011). Collagens play structural roles and contribute to mechanical properties, organization and pattern shaping of tissues. Proteoglycans are characterized by a core protein that is covalently linked to glycosaminoglycans (GAGs), which are long, negatively charged and linear chains of disaccharide repeats. The primary biological function of proteoglycans derives from the biochemical and hydrodynamic characteristics of the GAGs, which bind water to provide hydration and compressive resistance (Mouw et al., 2014). Glycoproteins are proteins, which often carry conventional asparagine-linked oligosaccharides with multiple functions, including promoting cell adhesion or migration in appropriate locations and signalling transduction to other cells (Hynes and Naba, 2012).

During the early development of animals, the ECM plays vital roles in providing structures, guiding migration and polarity of cells, maintaining morphogenesis and coherence of tissues (Brown, 2011). The ECM is synthesized and secreted by embryonic cells beginning at the very early stages of development (Rozario and DeSimone, 2010). Dysfunction of ECM components causes numerous developmental defects and diseases involving musculoskeletal, cardiovascular, renal, ocular and skin deficiency in mammals (Bateman et al., 2009; Lu et al., 2012). Therefore, characterizing the global composition and architecture of the ECM during embryogenesis could lead to important discoveries. Advances in high-throughput sequencing technology has allowed us to trace the ECM genes comprehensively and globally (Hynes and Naba, 2012; Naba et al., 2016).

Ascidians are the largest class within the subphylum Tunicata (Urochordata) in the chordate phylum. Ascidians of the genus *Ciona* are widely used model organisms for chordate developmental genomics because of their similar embryonic body plan to that of vertebrates (Stolfi and Christiaen, 2012). The genome of *Ciona intestinalis* and *Ciona savignyi* have both been sequenced (Dehal et al., 2002; Small et al., 2007). The experimental malleability and unique phylogenetic position of the sea squirt *Ciona* provides as an interesting model system to study the molecular composition and architecture of ECM in embryogenesis and larval metamorphosis. Most of the ECM genes in *Ciona* have already been characterized, such as collagen (Vizzini et al., 2002, 2008), decorin (Pavão et al., 1994), glypican (Mita et al., 2010), podocan (Park et al., 2008), syndecan (Chakravarti and Adams, 2006), leprecan (Capellini et al., 2008), agrin (Huxley-Jones et al., 2007), nidogen (Huxley-Jones et al., 2007), fibrillin (Jensen et al., 2012), fibulin (Cota et al., 2014), laminin (Oda-Ishii et al., 2010), SCO spondin (Ishibashi et al., 2005), tenascin (Tucker et al., 2006), thrombospondin (Adams et al., 2003), SPARC (Kawasaki et al., 2007), uromodulin (Kawashima et al., 2005) and von willebrand factor (Sasaki et al., 2003). A large number of phylogenetic trees of *Ciona* ECM genes have already been analyzed and published previously, such as *agrin* (Huxley-Jones et al., 2007), *perlecan* (Huxley-Jones et al., 2007), *collagens* (Aouacheria et al., 2004; Huxley-Jones et al., 2007), *thrombospondin* (Adams et al., 2003; McKenzie et al., 2006), *syndecan* (Chakravarti and Adams, 2006), *leprecan* (Capellini et al., 2008) and *tenascin* (Tucker et al., 2006). The spatial expression patterns of some ECM genes have been reported previously. For example, *laminin* showed a notochord specific expression at late neurula, mid-tailbud and late tail extension stages (Veeman et al., 2008). The expression of *glypican3/5* was in the anterior epidermis at gastrula stage (Mita et al., 2010). The expression of *leprecan* in *C. intestinalis* was confined to the notochord at tailbud stage (Capellini et al., 2008). *Collagen XVIII* and *collagen XI* were expressed in notochord, nerve cord, endodermal strand and endoderm during embryogenesis (Hotta et al., 2008). Functional analysis of the ECM component laminin (Veeman et al., 2008) and fibronectin (Segade et al., 2016) in *Ciona* has already revealed that they are essential for tissue intensity and organ formation. The expression of the dominant negative form of leprecan in notochord cells also resulted in the disruption of their linear, single-file arrangement with respect to the anterior-posterior axis (Dunn and Di Gregorio, 2009). However, the comprehensive distribution and expression dynamics of ECM genes during early development and metamorphosis of *Ciona* are still lacking, limiting our understanding of many processes in embryogenesis and tissue morphogenesis.

In this study, we first applied wheat germ agglutinin (WGA) staining to probe the ECM architecture of *Ciona* embryos. Scanning electron microscopy (SEM) and transmission electron microscopy (TEM) were also employed to obtain more precise structures of the ECM. Then, we used the RNA sequencing (RNA-seq) data from three *C. savignyi* embryo libraries at different stages to identify ECM genes and acquire their expression patterns. We have revealed the profile of molecular composition and architecture of the ECM in ascidian embryogenesis and larval metamorphosis. Our results will help to further understand the function of ECM in chordate development.

## RESULTS

### ECM structure examined by WGA staining and electron microscopy

**Fig. 1.**
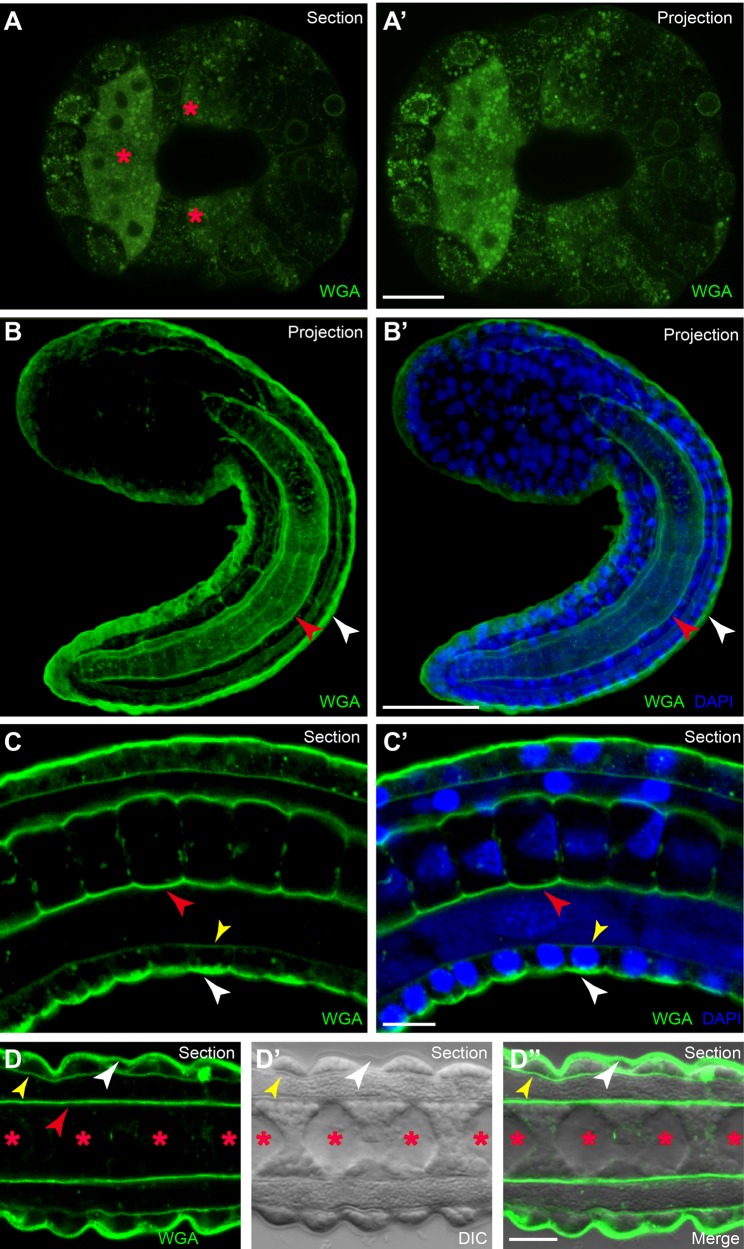
**ECM architecture of *Ciona* embryos as shown by WGA labelling.** (A,A′) Global view of WGA-stained embryos at gastrula stage. The red asterisks indicate notochord cells. (B,B′) Global view of WGA-stained embryos at tailbud stage. There are two distinct layers of ECM: the outer layer (white arrowheads) at the apical surface of epithelia, and an inner layer (red arrowheads) of sheath surrounding the notochord. (C,C′) Magnified tail part of WGA-stained early embryos. The yellow arrowheads in C and C′ indicate the weaker layer localized at the basement membrane of epithelia. (D-D″) Magnified view of the ECM structure in later stage embryos. The red asterisks indicate the apical lumen, which cannot be stained by WGA in later stage embryos. Scale bars: 100 µm in A′ and B′; 10 µm in C′ and D″.

The detailed architecture of the ECM structure in *Ciona* embryos was examined through WGA staining and electron microscopy, respectively. WGA contains a group of closely related isolectins and selectively binds to N-acetyl-D-glucosamine and N-acetylneuraminic acid (sialic acid) residue (Schwab et al., 1978). Fluorescently tagged WGA staining is one of the most widely used and suitable methods for detection, visualization and quantification of fibrotic or connective tissue of animals (Kostrominova, 2011). Although it has been used to detect the changes in lectin binding affinities in test cells and new cells during the swimming period of *C. intestinalis* larvae (Sato and Morisawa, 1999), a whole view of fluorescent WGA staining in *Ciona* embryos was lacking. Here we, for the first time, used a fluorescent WGA probe to display the ECM architecture in *Ciona* embryos. The results showed WGA-positive signalling distributed inside all cell types but not on the cell surface in gastrula embryos. Interestingly, WGA signalling in notochord cells was stronger than in other cells (red asterisks in Fig. 1A,A′). There were two distinct layers of ECM in tailbud embryos at 18 hours postfertilization (hpf) (Fig. 1B,C). The outer layer was thick, covering the apical surface of the epithelia (white arrowhead in Fig. 1B). The inner one was the notochord sheath (red arrowhead in Fig. 1B,B′). Except these two major layers, there existed a weaker WGA labelling layer localized at the basement of epithelia (yellow arrowhead in Fig. 1C,C′). At a later stage (21 hpf), an obviously visible ECM population appeared between the adjacent notochord cells in *Ciona* embryos, but could not be stained by WGA (red asterisk in Fig. 1D-D″). The tunic is a special feature of ascidians. It distributes in the outer layer of epidermis cells and can be labelled by WGA staining (white arrowhead in Fig. 1D-D″).

**Fig. 2.**
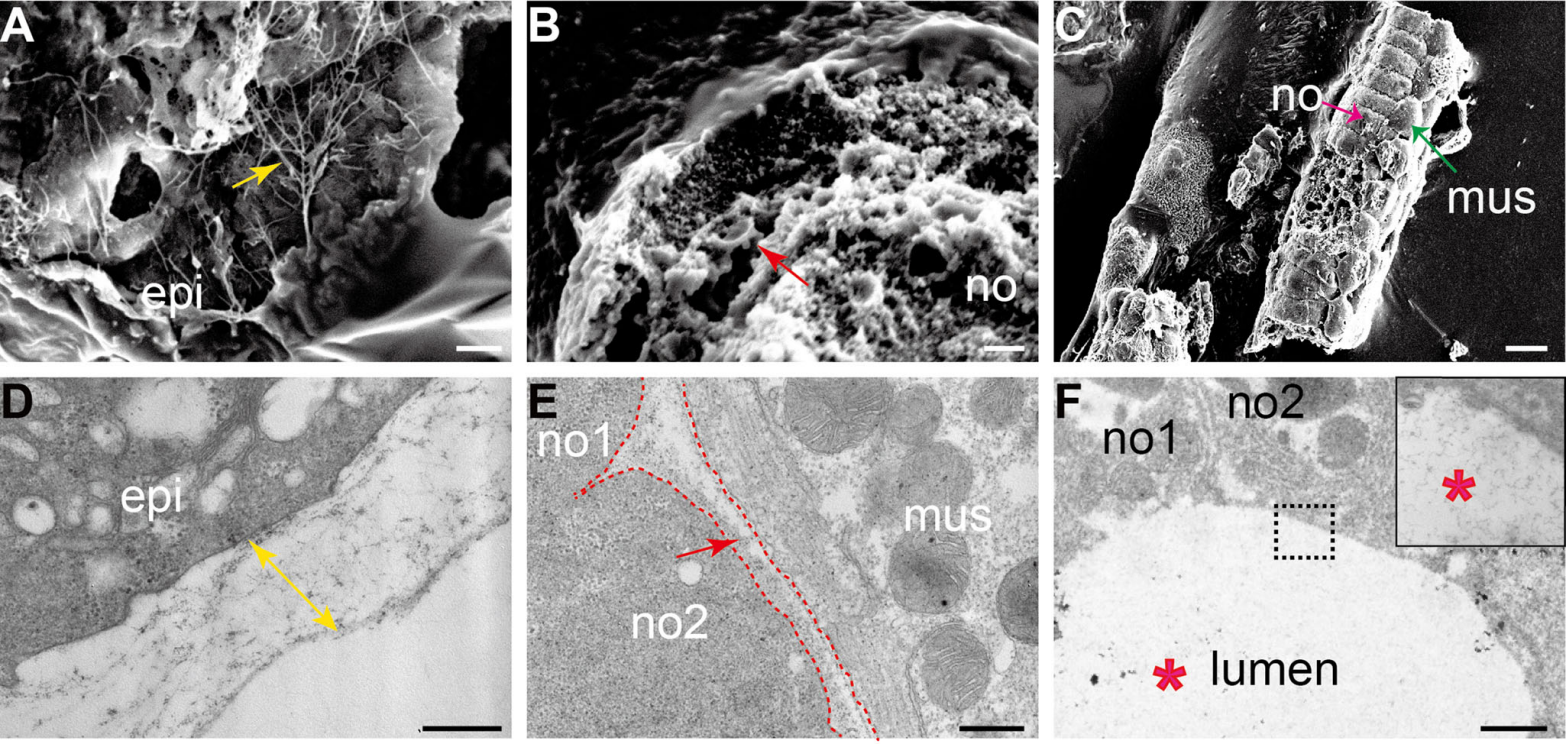
**ECM structures of *Ciona* embryos as shown by electron microscopy.** (A) SEM image of the body surface of tailbud stage embryos. The yellow arrow indicates separated short fibril structures. (B) Cross-section of a fragment of a tail part scanned by SEM. The red arrow indicates notochord sheath. (C) Longitudinal view of the tail part from tailbud embryos, as shown by SEM. The red arrow indicates linear notochord cells; the green arrow indicates the flank muscle cells. (D) The body surface of tailbud stage embryos, as shown by TEM. The bi-directional arrow indicates the thickness of the outer membrane. (E) Longitudinal section of the tail part, including the notochord and muscle cells. The red arrow indicates the fibrillar ECM in the notochord sheath. The red dashed line outlines the cell boundaries. (F) Cross section of the tail part including the notochord cells and central lumen. The red asterisks indicate low-density lumen. Inset shows a magnified view of the apical lumen. Scale bars: 10 µm in A and B; 100 µm in C; 500 nm in D and E; 1 µm in F. epi, epidermis cell; mus, muscle cells; no, notochord; no1, notochord cell 1; no2, notochord cell 2.

Electron microscopy was then used to reveal the detailed ECM structure and types. SEM revealed that single short fibrils existed on the split surface and along the edge of the torn membrane (yellow arrow in Fig. 2A). Its homogeneous structure was presumed to be made up of a tightly packed matrix. TEM showed that the outer membrane was relatively thick (bi-directional arrow in Fig. 2D). In notochord sheath, the ECM layer was not as thick as the outer membrane but more regular arranged (Fig. 2B,E). No separated fibrils were found on split cells like the outer membrane (Fig. 2C), suggesting that they are tightly organized, probably within a sheath structure. The apical ECM was secreted from notochord cells (Dong et al., 2009). TEM images revealed that it was low density with a large number of short chain structures (red asterisk in Fig. 2F), resembling GAGs (Hunziker and Schenk, 1984). This observation was consistent with a previous hypothesis that the notochord inner lumen contained viscous fluid (Dong et al., 2009).

Using WGA histological staining and TEM, we revealed that *Ciona* embryonic ECM mainly distributed in the outer membrane and notochord sheath in early embryos. At a later stage, during notochord tubulogenesis, the *de novo* formed pocket apical lumen appeared, eventually forming a single lumen inside the notochord.

### High-throughput RNA-seq data assembly and annotation

In this study, three *Ciona* embryonic and larval stages were chosen for RNA-seq and nine samples (three for each stage) were collected independently for biological replication. According to our observations, at 18 hpf [St. 24 (Hotta et al., 2007)], notochord cells elongated in a single line but the lumen did not appear; at 21 hpf [St. 25 (Hotta et al., 2007)], apical extracellular lumens appeared and expanded between adjacent notochord cells, later forming a notochord tube (Dong et al., 2009); at 42 hpf, the tail of the swimming larva disappeared in the process of metamorphosis.

Seven libraries were successfully constructed and sequenced including two 18 hpf samples, two 21 hpf samples and three 42 hpf samples. After removing adaptors and trimming low quality reads, the clean reads were obtained and then assembled into 147,212 transcripts and clustered into 110,279 unigenes. The all-unigenes, totalling 66 Mbp, with an average length of 599 bp and N50 length of 990 bp, were then used as references for annotation and expression analysis. By blast searching with a cutoff E-value <1e-5, 18,956 (17.18%) unigenes found putative homologues in the nr protein database from the NCBI, 7179 (6.5%) unigenes found putative homologues in the nt database and 15,184 (13.76%) unigenes found putative homologues in the Swiss-Prot database. The best aligning results were used for identification of ECM genes. KO, PFAM, gene ontology (GO) and KOG annotation were also conducted. In total, 25,637 (23.24%) unigenes were successfully annotated.

### Identification and expression pattern analysis of ECM genes

Among the annotated unigenes, 115 were identified as 67 types of ECM genes and divided into three groups. The first group was the collagen family with 21 unigenes corresponding to 14 homologue collagen genes of *C. intestinalis* (Table S1). The second group was the proteoglycan family with 10 unigenes corresponding to nine homologue genes, including syndecan, glypican-5, glypican-6, decorin, podocan, chondroadherin, neurocan, leprecan and perlecan (Table S2). In addition, nine unigenes corresponding to seven GAGs synthase genes were also identified, including chondroitin sulfate synthase, uronyl 2-sulfotransferase, heparan sulfate N-deacetylase and chitin synthase (Table S3). The third group was the glycoprotein family with 84 unigenes corresponding to 44 homologue genes, such as fibronectin, laminin, tenascin, nidogen etc. (Table S4). The phylogenetic trees of chondroadherin and slit2 are shown in Fig. S1.

To acquire the expression profile and identify differentially expressed genes (DEGs), we calculated the FPKM (fragments per kilo bases per million fragments) value and then used it to compare the expression differences between different samples (18-21 hpf, 21-42 hpf and 18-42 hpf). DEGs were screened with adjusted *P*<0.05. Among the 115 unigenes, which were annotated as ECM genes, 77 showed dynamic changes (upregulated or downregulated) between different stages (Tables S1-S4). For collagen family genes, most of the unigenes (18 out of 21) have dynamic expression patterns (Table S1). Among these genes, c119589g1 [collagen alpha-1 (II)], c117429_g1 [collagen alpha-1 (IV)], c120533g1 [collagen alpha-1 (V)], c118690_g1 (collagen type IX) and c119505g1 [collagen alpha-1 (XXVII)] were highly expressed at both 18 and 21 hpf embryonic stages, while c115706_g1 [collagen alpha-6 (VI)] and c118728_g2 [collagen alpha-1 (XXVIII)] were highly expressed at metamorphic larval stage (42 hpf) (Table S1). For the proteoglycan family, only c120275_g1 (chondroadherin) showed a high expression level at 18 hpf stage (Table S2). For GAG synthesis genes, chondroitin sulfate synthase 1 was highly expressed at 21 hpf stage (Table S3). For glycoproteins, c120356_g1 and c120356_g2 (nidogene-2) were highly expressed at both 18 and 21 hpf stages (Table S4).

**Fig. 3.**
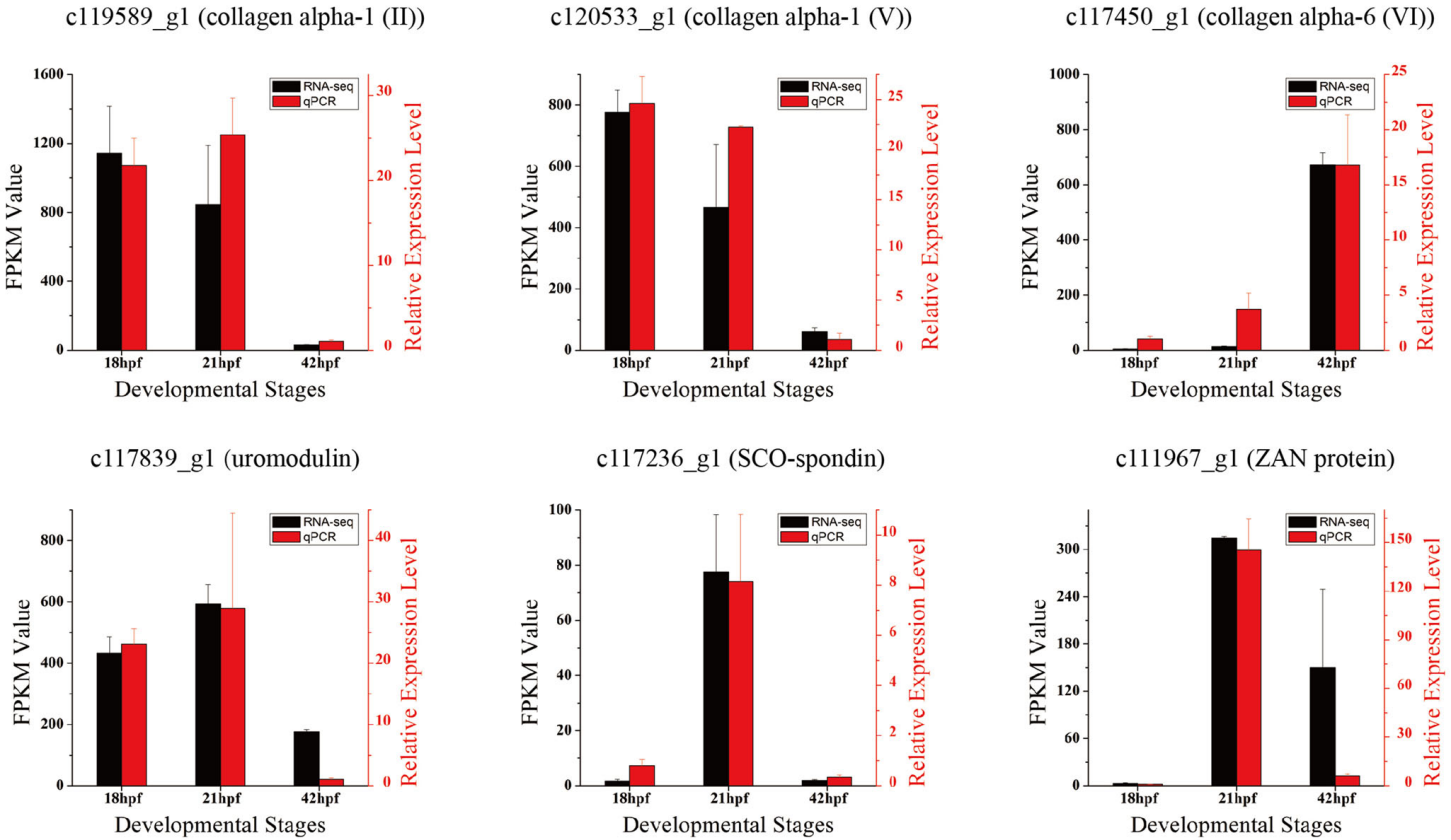
**Expression profiles of selected ECM genes from qPCR and RNA-seq.** Three developmental stages are shown in the *x*-axis. Data are mean±s.e.m. relative expression levels. RNA-Seq results are shown in black; qPCR results are shown in red. The expression profiles of the RNA-seq and qPCR data were consistent.

To confirm the expression level of annotated ECM genes, quantitative real-time polymerase chain reactions (qPCRs) were utilized to validate their expression patterns. Six ECM genes were selected for validation. The results showed that the expression profiles of the RNA-seq and qPCR data were consistent (Fig. 3).

**Fig. 4.**
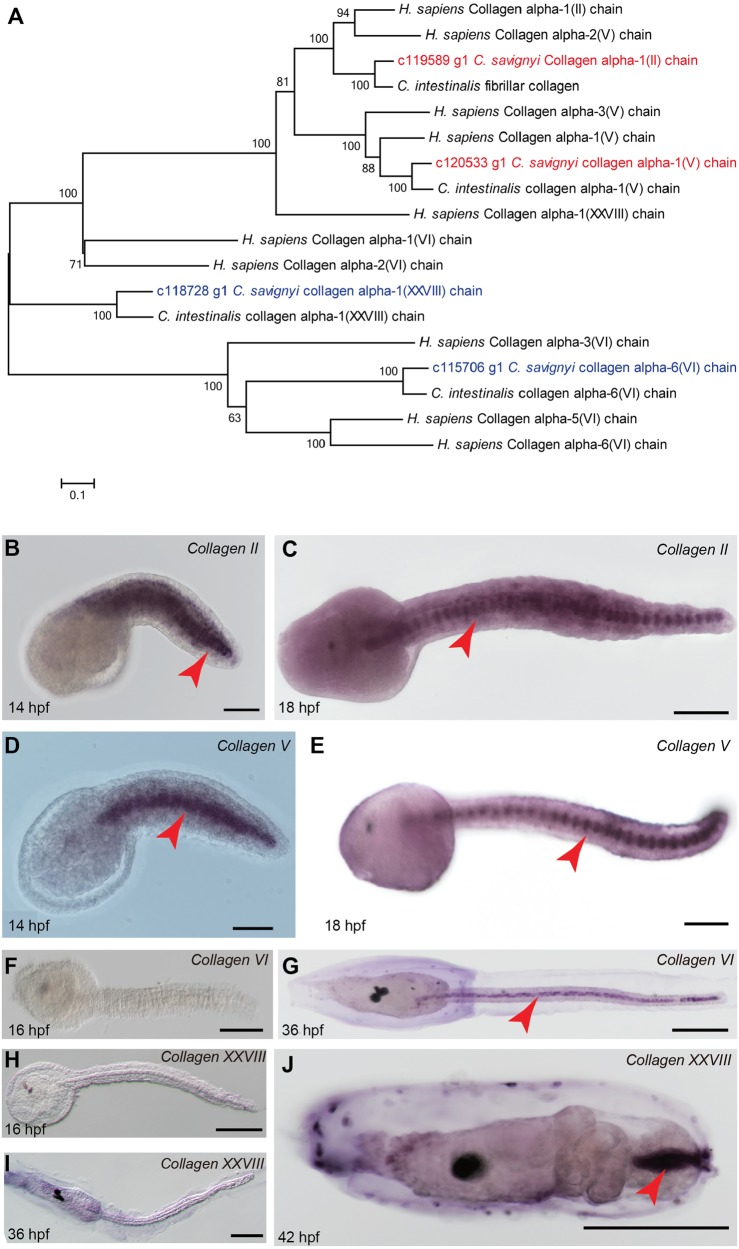
**Phylogenetic tree analysis of selected collagens and their expression patterns.** (A) The phylogenetic tree of collagens. The putative fibrillar collagens of *C. savignyi* are shown in red; the putative nonfibrillar collagens of *C. savignyi* are shown in blue. (B-J) Whole-mount *in situ* hybridization of c119589g1 [collagen alpha-1 (II)], c120533g1 [collagen alpha-1 (V)], c115706g1 [collagen alpha-1 (VI))] and c118728g1 [collagen alpha-1 (XXVIII)]. The red arrowheads indicate the signals in the different staged embryos. Scale bars: 100 µm.

In order to confirm the type of collagen genes with dynamic expression level in *C. savignyi*, a phylogenetic tree was built according to their putative protein sequence (Fig. 4A). In mammals, collagens can be subdivided into subfamilies based on their domain homology and supramolecular assemblies, including fibrillar, network-forming, FACITs, MACITs, anchoring fibrils, beaded-filament-forming and multiplexin collagens (Shoulders and Raines, 2009). In this study, c119589g1 [collagen alpha-1 (II)] and c120533g1 [collagen alpha-1 (V)] were classified as fibrillar collagens, while c115706g1 [collagen alpha-6 (VI)] and c118728g1 [collagen alpha-1 (XXVIII)] were classified as nonfibrillar collagens. To further examine their spatial expression, we performed whole-mount *in situ* hybridizations, and the results showed that c119589g1 [collagen alpha-1 (II)] and c120533g1 [collagen alpha-1 (V)] were specifically expressed in notochord cells at late tailbud stage (Fig. 4B-E), while c115706g1 [collagen alpha-6 (VI)] and c118728g1 [collagen alpha-1 (XXVIII)] did not express in 16 hpf embryos (Fig. 4F,H). In later staged larvae, collagen alpha-6 (VI) specifically expressed in notochord cells (red arrowhead in Fig. 4G). Collagen alpha-1 (XXVIII) did not express (Fig. 4I), but it presented in the posterior part of the metamorphic larval tail (red arrowhead in Fig. 4J).

## DISCUSSION

### ECM architecture in *Ciona* embryos and larvae

The ECM is a collection of extracellular molecules secreted by cells that provides structural and biochemical support to the surrounding cells. The modular domain structure of ECM proteins and their genes has allowed extensive exon or domain shuffling during evolution to generate hundreds of ECM proteins (Hynes, 2012). The animal ECM includes the interstitial matrix and the basement membrane. Interstitial matrix is present between various animal cells. Gels of polysaccharides and fibrous proteins fill the interstitial space and serve as a compression buffer against the stress placed on the ECM. Basement membranes are sheet-like depositions of ECM on which various epithelial cells rest (Theocharis et al., 2016).

### ECM function in embryogenesis and larval metamorphosis

ECM is composed of collagens, glycoproteins and proteoglycans assembled into a supramolecular meshwork that provides structural support, organization and orientation to tissues (Bosman and Stamenkovic, 2003). As well as being a substrate for cell growth, the ECM also influences many cell behaviours such as migration, proliferation, adhesion and differentiation. The composition of ECM and therefore the regulation of ECM degradation and remodelling serve pivotally in the control of embryogenesis of chordates (Davis and Senger, 2005).

Fibrillar collagen genes [collagen alpha-1 (II) and collagen alpha-1 (V)] were highly expressed at both 18 and 21 hpf stage. Considering the fibrillar structures in notochord sheath, they were most likely the constitution of notochord sheath. The arrangement of collagen fibrils within the notochord sheath was assumed to play a decisive role in determining its functional properties as a hydrostatic skeleton (Stemple, 2005).

Transmembrane cell adhesion receptor mua-3 was the most highly expressed genes in all three stages (Table S3). It is a kind of glycoprotein, which is essential for the formation of elastic fibres found in connective tissue (Kielty et al., 2002). As we can find many separated microfibrils on outer membrane by EM observation, collagen alpha-1 (IV), collagen type IX and transmembrane cell adhesion receptor mua-3 were probably the main constituent of the outer membrane in *Ciona* embryos. However, elastin, the important constituent of vertebrate skin was not identified in our dataset.

Except for structural roles, cell adhesion, cell-cell communication and differentiation are also common functions for ECM. For example, the matrix glycoprotein fibronectin has long been considered a vertebrate-specific gene, playing a major instructive role in vertebrate embryonic development (Singh et al., 2010). Targeted knockdown in the notochord lineage indicates that fibronectin is required for proper convergent extension in *C. intestinalis* (Segade et al., 2016). Laminin-mediated boundary formation also can drive considerable tail elongation in *Ciona* (Veeman et al., 2008).

Several ECM genes were highly expressed at 42 hpf stage, such as collagen alpha-6 (VI) and collagen alpha-1 (XXVIII), which were speculated to be associated with tail regression during larval metamorphosis.

### ECM in notochord lumen formation and expansion

In this study, we collected the embryos of *C. savignyi* at late tailbud stage (18 and 21 hpf) and metamorphic larval stage (42 hpf) for high throughput RNA-seq. At 18 hpf stage, the ascidian notochord formed a linear cord without apical lumen, which subsequently presented at the interface of adjacent notochord cells at 21 hpf stage. The DEGs between 18 and 21 hpf were possibly related to lumen formation.

Recently, the roles of ECM during lumen initiation have gained increasing attention. ECM scaffolding can guide lumen elongation by inducing anisotropic intercellular mechanical tension (Li et al., 2016). The ECM proteins FRAS1 and nidogen-2, which are highly expressed at 18 hpf stage and have been demonstrated to be involved in the regulation of embryonic development (McGregor et al., 2003; Salmivirta et al., 2002), might play roles in notochord lumen initiation.

Hemicentin 1, uromodulin and chondroitin sulfate synthase 1 were highly expressed genes at 21 hpf. The notochord lumen was presumed to be hydrostatic by electron microscopy observation. We therefore speculated that hemicentin 1, uromodulin and chondroitin sulfate were the components of notochord lumen. During notochord development in ascidian, ECM was synthesized and deposited on both the apical and basal sides. On one hand, the ECM provides the initial cue that orients the apical-basal polarity axis (O'Brien et al., 2001). On the other hand, the accumulation of secreted or membrane-anchored ECM via exocytosis in the initial lumen may act as a ‘sponge’ to drive the influx of fluid (Denker and Jiang, 2012). In addition, WGA signalling was not found in the lumen. We therefore speculated that no WGA ligands exist in *Ciona* notochord lumen.

Using high-throughput RNA-seq and bioinformatics analysis, we revealed the abundance and dynamic expression of ECM genes. We then conducted WGA staining and electron microscopy to obtain the detailed ECM architecture of embryos. The ECM might be involved in additional cellular processes such as cell migration and differentiation during ascidian development. The mechanisms underlying the regulation of ECM expression remain undefined and require further research.

## MATERIALS AND METHODS

### Embryo sampling

Adult *C. savignyi* were collected from Qingdao, China. Eggs were first removed and then mixed in seawater with sperm from other individuals. After fertilization, the embryos were cultured at 16°C. In this study, we collected the embryos at 18, 21 and 42 hpf for RNA extraction and high-throughput sequencing. Nine samples were collected independently (three for each stage), with ∼1000 embryos in each sample.

All of the procedures involved in the handling and treatment of *C. savignyi* were approved by the Ocean University of China Institutional Animal Care and Use Committee (OUC-IACUC) prior to the initiation of the study. All experiments and relevant methods were conducted in accordance with the approved guidelines and regulations of the OUC-IACUC.

### Staining and imaging

Dechorionated embryos were fixed with 4% paraformaldehyde in seawater. Fixed embryos were stained with WGA and DAPI. To prepare 1 mg/ml stock solution, WGA (Alexa Fluor 488 conjugate, Molecular Probes, Eugene, USA) was dissolved in millipore water. The working concentration was 1 μg/ml. After washing four times with PBST, embryos were stained with WGA (1/100) overnight at 4°C. Differential interference contrast (DIC) images were taken with a Ni microscope (Nikon Instruments, Tokyo, Japan). Confocal images were taken with an A1R confocal laser-scanning microscope (Nikon Instruments). Image analysis and three-dimensional reconstruction were performed using Nikon NIS software packages. For electron microscope studies of the notochord, the samples were fixed with 1% glutaraldehyde in 80% seawater and then dehydrated and observed with a S-3400N scanning electron microscope (Hitachi, Tokyo, Japan) and a H-7000 transmission electron microscope (Hitachi).

### RNA extraction and high-throughput sequencing

Total RNA was extracted using RNAiso plus reagent (Takara, Shiga, Japan), following the manufacturer's instructions. The RNA was dissolved in 30 μl water. The concentration of each sample ranged from 66 to 368 ng/μl. RNAs were assessed by electrophoresis in 1% agarose gel and quantified using a NanoDrop 1000 spectrophotometer (Thermo Fisher Scientific) and Agilent 2100 Bioanalyzer (Agilent Technologies, Santa Clara, USA). RNA purification, reverse transcription, library construction and sequencing were conducted by Novogene (Tianjin, China). Seven samples were constructed successfully and sequenced using HiSeq 2500 (Illumina, San Diego, USA).

### Sequencing data assembly, annotation and bioinformatics analysis

The raw reads from seven samples were preprocessed by removing adaptors, and those low quality reads were also excluded in subsequent analysis. The clean reads of each stage were then assembled into unigenes using the Trinity program (Grabherr et al., 2011). In order to annotate all-unigenes, blast alignments (Altschul et al., 1990) (E value, 1e-5) against the Nr, Nt, Swiss-Prot, KEGG, and COG databases were performed. GO analysis was conducted using BLAST2GO program (Conesa et al., 2005).

By means of reads mapping to unigenes, the FPKM value of unigenes in each sample were obtained and used for comparing the expression difference between samples. We chose those with adjusted *P*<0.05 as DEGs by DEseq analysis (Anders and Huber, 2010).

### Validation by qPCR and whole-mount *in situ* hybridization

qPCR analysis was used for validation, with SYBRGreen used as the DNA-binding fluorescent dye and 18S rRNA gene used as an internal standard. Relative gene expression levels were calculated using the comparative Ct method with the formula 2^−ΔΔCt^ (Livak and Schmittgen, 2001). The qPCR results were then compared with transcriptome data (FPKM value) to detect the expression correlation of each gene. Dechorionated embryos were fixed with 4% paraformaldehyde. Whole-mount *in situ* hybridization was carried out essentially as previously published (Christiaen et al., 2009), using a hybridization temperature of 55-57°C.
